# Phospholipid transfer by ERMES components

**DOI:** 10.18632/aging.101434

**Published:** 2018-04-28

**Authors:** Toshiya Endo, Yasushi Tamura, Shin Kawano

**Affiliations:** 1 Kyoto Sangyo University, Faculty of Life Sciences and Institute for Protein Dynamics, Kamigamo-motoyama, Kyoto, Japan

**Keywords:** mitochondria, ER, inter-organelle contact, ERMES, phospholipid transfer

Mitochondria are crucial for production of cellular energy ATP, metabolism of lipids, amino acids, and iron, and cellular decision to terminate its activities, apoptosis. However, mitochondria do not function alone, but cooperate with other organelles to meet the cellular demands and extracellular signaling, through physically apposed structures called inter-organelle contacts. Inter-organelle contacts are found for multiple pairs of organelles, and likely mediate direct exchange of metabolites and information between organelles. In particular, since phospholipid biosynthetic pathways encompass the ER and mitochondria, which are not linked with each other by vesicular trafficking, exchange of intermediate phospholipids through inter-organelle contacts should be essential for cellular phospholipid supply. Aberrant inter-organelle contacts between the ER and mitochondria are connected to human neurodegenerative diseases, as well [1].

ERMES (ER-mitochondrial encounter structure) between the ER and mitochondria in yeast is one of the best characterized inter-organelle contacts [2]. ERMES contains four core subunits, an N-anchor ER membrane protein, Mmm1, a β-barrel mitochondrial outer membrane (OM) protein, Mdm10, an OM protein without an obvious transmembrane segment, Mdm34, and a peripheral OM protein, Mdm12. ERMES was proposed to facilitate lipid exchange between the ER and mitochondria [2], and Mmm1, Mdm12, and Mdm34 contain a synaptotagmin-like mitochondrial-lipid-binding protein (SMP) domain, which forms a hydrophobic cavity and likely binds to hydrophobic molecules such as phospholipids. However, it is not clear if SMP domains of the ERMES subunits are able to transfer lipids between membranes efficiently, and if this is the case, it remains open how hydrophobic lipid molecules are transported between the organelles via ERMES by crossing the aqueous cytosol.

In a recent study, we reported the X-ray structures of the *Kluyveromyces lactis* Mdm12 SMP domain (Mdm12) and *in vitro* lipid transfer activities of Mdm12, the SMP domain of Mmm1 (Mmm1) fused to maltose binding protein (MBP-Mmm1), and the Mmm1-Mdm12 complex (Mmm1-Mdm12) [3]. The 2.25 Å resolution structure of lipid-bound Mdm12 exhibited an inverted cone-like overall shape and the presence of a deep hydrophobic pocket along the cone axis with an opening at the base, which accommodates a phospholipid molecule in a tail-in manner. The structures of the Mdm12 SMP domains from various organisms and identification of phospholipids in their hydrophobic pockets in our and other groups’ studies [3–7] established that Mdm12 is a lipid-binding protein.

We then tested lipid transfer abilities of Mdm12, MBP-Mmm1, and Mmm1-Mdm12 by using lipid liposomes *in vitro* [3]. Purified free Mdm12 alone or Mmm1 as a purified fusion protein MBP-Mmm1 was capable of extracting phospholipid from donor liposomes and transferring it to acceptor liposomes, but their lipid transfer activities between liposomes were very low. On the other hand, the lipid transfer activity was significantly enhanced when Mdm12 and Mmm1 formed a hetero-oligomeric complex with each other. The *in vitro* lipid transfer activity of the Mmm1-Mdm12 complex was impaired by mutations in the hydrophobic pockets of Mmm1 or Mdm12. Furthermore, these Mmm1 and Mdm12 mutations were found to impair the ERMES-dependent phosphatidylserine transport from the ER to mitochondria in the *in vitro* lipid transport assays with isolated mitochondria and ER membranes [3]. These results indicate that the lipid transfer activity of the Mmm1-Mdm12 complex between membranes arises from the cooperation of Mmm1 and Mdm12, and in other words, the Mmm1-Mdm12 complex functions as a minimal unit mediating lipid transfer between the membranes by the ERMES.

**Figure 1:**
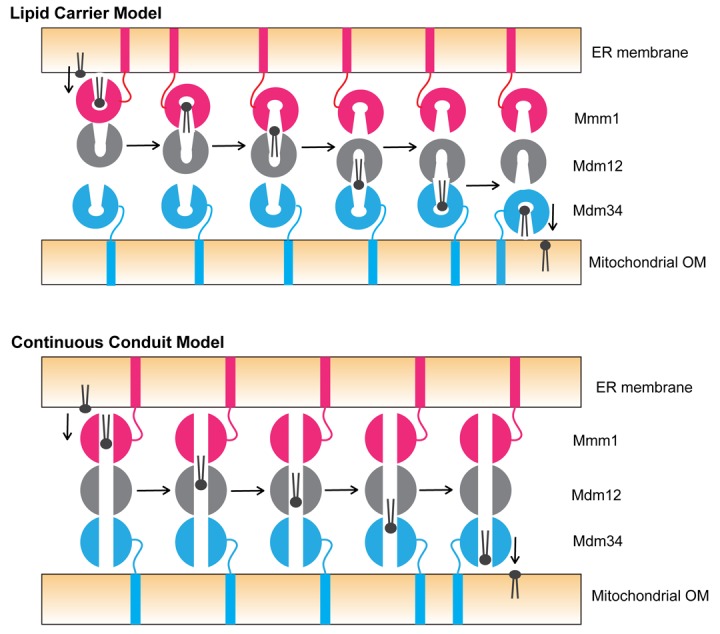
**Models of phospholipid transfer by ERMES between the ER and mitochondrial OM.** (Upper) Lipid carrier model. Mmm1 may change the relative geometry to Mdm12 from the tail (Mmm1)-to-head (Mdm12) contact to the head (Mmm1)-to-head (Mdm12) contact, so that the phospholipid molecule bound to the hydrophobic pocket of Mmm1 can be transferred to the one of Mdm12 through the outlets of the both pockets at the head-to-head interface between Mmm1 and Mdm12. Upon receiving the phospholipid molecule from Mmm1, Mdm12 may switch the partner from Mmm1 to Mdm34, with changing the relative geometry to Mdm34 from the tail (Mdm12)-to-head (Mdm34) to the head (Mdm12)-to-head (Mdm34) contact for further lipid transfer. (Lower) Continuous conduit model. The phospholipid molecule extracted by Mmm1 from the ER membrane may diffuse through the hydrophobic conduit from Mmm1 to reach Mdm34 via Mdm12, and then is inserted into the mitochondrial OM by Mdm34.

Now the key question is how ERMES facilitates phospholipid transfer between the membranes. Since Mmm1 has an ability to extract lipid from and insert lipid into the ER membrane [3], it may function as an extractor or inserter of lipids in the ER membrane. Likewise, Mdm34 as an SMP domain-containing protein, may play a similar role in the mitochondrial OM. Then the peripheral membrane protein Mdm12 could mediate lipid transfer between Mmm1 and Mdm34. For the transfer of lipid from Mmm1 to Mdm34 via Mdm12, two possible models can be considered (Fig. 1). In the lipid carrier model, Mmm1, Mdm12, and Mdm34 change their orientations and positions relative to the other components to put the outlet of the lipid-binding pocket of one component close to the one of the other component for efficient transfer of lipid molecules. The continuous conduit model assumes the presence of a continuous hydrophobic tunnel running from Mmm1 to Mdm34 via Mdm12 for processive movement of lipid molecules between Mmm1 and Mdm34. However, the lipid carrier model is not consistent with the stable complex formation between Mmm1 and Mdm12, without any dynamic exchange with their free forms [3]. The continuous conduit model is not supported by our finding that the bottom of the hydrophobic pocket of Mdm12 is tightly closed [3], and by the recently reported X-ray structure of the complex of the Mmm1 and Mdm12 SMP domains from different organisms, which does not show the presence of a continuous conduit wide enough for efficient lipid transfer between Mmm1 and Mdm12 [7]. Clearly, both models point to the requirement of dynamic nature of the conformations of and interactions among SMP domain-containing proteins of ERMES for lipid transfer between the ER and mitochondria, which would also give an insight into the mechanism of lipid transfer through SMP domain-containing proteins at other inter-organelle contacts like the ER-plasma membrane contact sites and NVJ (nuclear-vacuole junction).
